# Casting Doubt on the Role of Mitochondria in Tumorigenesis

**DOI:** 10.1371/journal.pmed.0020373

**Published:** 2005-10-04

Mitochondrial DNA (mtDNA) has been intensively studied over the past two decades, and point mutations, more commonly known as deletions, of this DNA are known to be involved in several syndromes. Unlike nuclear DNA, with 46 chromosomes, half from each parent, mtDNA is just one piece of genome of which there are many copies, but all copies come only from the mother. Mitochondrial disease syndromes, such as MELAS, have a range of different clinical manifestations depending on how many copies of the abnormal mtDNA are present in affected cells.

There are several international resources of mtDNA sequences. From these sequences, it has been possible to show that different population groups have different patterns of substitutions in the mtDNA—so-called haplogroups; this information has been used, for example, in the investigation of the origin and migration patterns of human populations, and some investigators have even suggested that it could be used to trace back to earliest human history the founding mothers of humanity.

More recently, however, attention has turned to the question of whether mtDNA is involved in tumor formation. However, deciding whether mutations are harmful or innocuous has been difficult. One concern is that isolation of mtDNA from any tissue is not simple, and may be particularly difficult from tumor samples, which are often contaminated with exogenous DNA.

In a hard-hitting paper, Antonio Salas and colleagues cast more doubt over a causal role for mtDNA alterations in tumors; they have now reassessed many of the studies that have examined the role of mtDNA in tumorigenesis, and concluded that much of the data are at best questionable. The group used a phylogenetic approach to analyze the reported work, which compared the sequences under consideration with the current database of complete sequences of mtDNA. The authors believe that such an approach is essential to look at the overall picture of the mtDNA rather than assessing each substitution in the mtDNA independently.

Salas and colleagues conclude that more than 80% of published mtDNA sequencing studies contain obvious errors, and that many of the published results that implicate mutations in tumorigenesis are in fact part of normal population variability, and their presence must be due to contamination of the tumor sample. Salas does not hold back in his criticisms. He states that the result of such sequencing “disasters” is that flawed results are not filtered out from the clinical literature, which makes the task of interpreting the role of mtDNA in the tumor process very difficult. [Fig pmed-0020373-g001]

**Figure pmed-0020373-g001:**
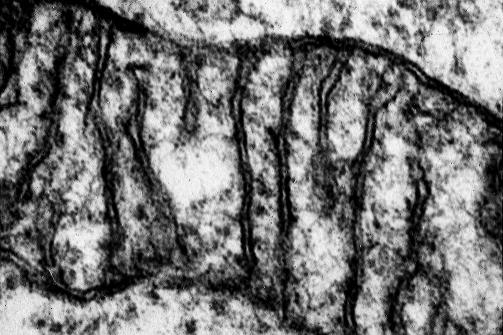
The Role of Mitochondria in Tumorigenesis is not yet clear

The researchers draw analogies to the time when ancient DNA sequencing was beginning, and many contaminated samples were amplified and claimed to yield prehistoric DNA. They urge clinical geneticists to use checks similar to those proposed to assess authenticity for ancient DNA studies: special care should be taken in sequencing and documentation, raw sequence data should be made fully accessible to referees and readers, and the complete record of data from the population genetics field should be used to put the results in context.

The group's findings will undoubtedly cause much debate in this research community. With their conclusion that “there is no precedent that we know of in the genetics literature for such a high number of flawed papers (most of them published in high-rank journals), which affect a whole subfield of clinical research,” they urge reconsidering the role of mtDNA in tumorigenesis.

